# Helping behaviours of community members towards older adults and the related factors: a cross‐sectional study

**DOI:** 10.1111/psyg.13050

**Published:** 2023-11-21

**Authors:** Ayumi Igarashi, Hiroshige Matsumoto, Haruno Suzuki, Manami Takaoka, Haruna Kugai, Mariko Sakka, Noriko Yamamoto‐Mitani

**Affiliations:** ^1^ Department of Gerontological Home Care and Long‐term Care Nursing, Division of Health Sciences & Nursing, Graduate School of Medicine The University of Tokyo Tokyo Japan; ^2^ Department of Community Health Nursing, Division of Health Sciences & Nursing, Graduate School of Medicine The University of Tokyo Tokyo Japan; ^3^ Department of Adult Nursing, Faculty of Nursing Kawasaki City College of Nursing Kanagawa Japan; ^4^ The Faculty of Medicine University of Tsukuba Ibaraki Japan

**Keywords:** aged, community support, dementia, helping behaviour

## Abstract

**Background:**

Community members can play important roles in helping older adults in their community. This study aimed to clarify the actual situation of community members' helping behaviours towards older adults and examine the related factors.

**Methods:**

This cross‐sectional study was conducted using an online survey system with a sample of 1000 community members in the Tokyo metropolitan area selected using quota sampling. Participants were asked about their experiences with helping an older adult, involvement with older adults with dementia, knowledge of dementia and care resources in the community, and perceptions regarding the community. Content analysis was used to classify participants' freely answered responses about helping behaviours, with logistic regression analysis subsequently used to examine the related factors.

**Results:**

Community members provided older adults with various types of spontaneous help, including help with walking (20.0%), accident care (16.8%), giving directions to a destination (11.6%), accompanying them to a destination (12.9%), and support in daily life (10.4%). In the multinominal logistic regression analysis, advanced helping behaviours were associated with having a family member with dementia, experiences involving people living with dementia, knowledge of dementia and community support centres, and a stronger sense of community integration (*P* < 0.05). The reasons for not being able to help included being physically unable to (42.5%), not feeling responsible (19.3%), not knowing how to help (17.4%), and hesitating to help (14.4%).

**Conclusion:**

The results suggest that providing learning opportunities for community members could further promote their helping behaviours for older adults. These could include interacting with older adults, especially those living with dementia; promoting a sense of community integration; or receiving training in helping actions. Such efforts could support the development of an effective community‐based care system for older adults.

## INTRODUCTION

In ageing societies worldwide, efforts have been made to realise ‘ageing in place’, which aims to enable older adults to live in their own communities as long as possible. As an international initiative, the development of age‐friendly communities/cities (AFCCs) ‘encourages active ageing by optimising opportunities for health, participation and security in order to enhance quality of life as people age’^1^ and has been promoted to realise ageing in place.^2^ In Japan, the government has promoted a community‐based integrated care system^3^ aimed at managing AFCCs. In this system, it is expected that various community members, including those other than health/social care professionals, will support older adults in the community. Therefore, individual community members should play a role in providing help to older adults.

In previous literature, the helping behaviours of community members have been classified as ‘formal planned helping’ (e.g., donating money, serving as a volunteer), ‘informal planned helping’ (e.g., looking after a sick friend, giving a ride to a friend without transportation), or ‘spontaneous helping’, defined as ‘helping that you provide immediately, with little or no prior thought’ (e.g., giving directions to a stranger, providing assistance to a stranger who has fallen over).^4^ Under the notion of a ‘community‐based integrated care system’, municipalities and community health/social care professionals have made efforts to build community networks to support older adults in the community (e.g., a system for monitoring older adults living alone, and mutual help among neighbours^5^, ^6^). This aims to promote ‘formal’ or ‘informal’ planned helping among community members.

At the same time, community members might also be compelled to provide spontaneous as well as planned help, since older adults with physical and/or cognitive disabilities can face sudden difficulties in their daily lives, such as falling or getting lost. If, in addition to planned behaviours, people develop the ability to spontaneously help older adults, it could further strengthen community‐based integrated care systems. To consider strategies for promoting spontaneous helping behaviours among community members, it is necessary to clarify the nature of these helping behaviours for older adults and identify the related factors.

Few studies have investigated spontaneous helping behaviours for older adults in the community. Most previous studies have focused specifically on planned helping behaviours for people living with dementia (PLWD). Several studies have revealed that community members' knowledge of dementia and/or interaction with PLWD was associated with positive attitudes towards PLWD and the intention to help.^7^, ^8^, ^9^, ^10^ Experiences of caring for PLWD were associated with positive attitudes towards PLWD in nursing students^11^ and a person‐centred care approach in healthcare professionals.^12^ Interventions have also been provided to improve attitudes and helping behaviours towards PLWD. Attending a dementia supporter training program, which is a standardised Japanese dementia education program, improved confidence in nursing students and nurses in caring for PLWD.^13^ After a training program including interaction with PLWD, volunteers' knowledge of dementia and attitudes towards PLWD improved,^14^ and after a service‐learning program that involved interaction with older adults, university students' comfort in working with adults with cognitive impairment significantly improved.^15^, ^16^ Furthermore, the psychological sense of community—the extent to which people feel connected to a community—predicted the concurrent and future volunteerism of community members.^17^

Thus, community members' knowledge of support for PLWD, experience in caring for or being involved with PLWD, and sense of community could affect also spontaneous helping behaviours towards older adults, including those without dementia. Therefore, this study aimed to clarify the actual situations of spontaneous helping behaviours of community members towards older adults and examine related factors such as knowledge of support, involvement with PLWD, and community perceptions.

## METHODS

### Sample and design

A survey was conducted to understand the need to develop educational programs for PLWD. The survey participants were recruited from the registered members of an internet research company (Rakuten Research). They were aged 15–69 years and were distributed across nine cities in the Tokyo Metropolis. The quota‐sampling method was based on 11 participants for each stratum based on city (nine cities), age (six age groups: teens to sixties), and sex (two strata). If a stratum contained fewer than 11 participants, it was supplemented with participants from an adjacent age group; in particular, there was a limited number of registered members aged 15–19 years, meaning that only 10 participants were recruited from that age group. Finally, 1000 participants were recruited for this study. Survey responses were voluntary, and respondents were given rewards by the survey company.

### Measurements

In the questionnaire, participants were asked about their demographics and their experience with helping older adults. To understand the factors related to helping behaviours, we also asked about their involvement with PLWD, knowledge of care, and perceptions of the community.

#### Demographics

The characteristics of the participants included age, sex, living arrangements, duration of living in the current community, and whether they worked as health or social care professionals.

#### Experiences of support and inability to support older adults

First, the participants were asked about their experience with helping or not being able to help an older adult other than their family in the community who faced difficulties. Those who had such experiences were asked to describe them as open‐ended responses. Participants with the experience of helping an older adult were asked to describe (i) what difficulty the older adult faced and (ii) how they helped. Those with experience of not being able to help were asked to describe (i) what difficulty the older adult faced and (ii) why they could not help. If they had more than one helping or non‐helping experience, they were asked to describe the one most impressive to them.

#### Involvement with a person with dementia

To measure involvement with a PLWD, participants were asked whether they had any family members with dementia and whether they had any experience interacting with a PLWD other than a family member.

#### Knowledge of care: dementia and community resources for support

Knowledge of care was measured by experience with attending a dementia supporter program^18^ and knowledge of dementia and community general support centres.

Knowledge of dementia was measured using a scale developed and validated by Mikami *et al*.^19^ This scale comprises 10 statements; for example, ‘Dementia is mainly managed through inpatient treatment’, ‘Some forms of dementia are associated with shivering and stiffness of the limbs’, and ‘Appropriate interaction with PLWD can relieve the symptoms of dementia’. In this study, the participants responded to each question with ‘yes’ or ‘no’, and the number of correct answers was summed (range: 1–10) for each participant. Higher scores indicated greater knowledge.

#### Perceptions of the community

To understand the participants' perception of the community, we measured ‘a sense of community’. This includes the feeling that members have of belonging—that members matter to one another and to the group—and a shared faith that members' needs will be met through their commitment to being together.^20^ Sense of community was measured using the short version of the Sense of Community scale,^21^, ^22^ which was rated using a 5‐point Likert‐type scale ranging from 1 (*disagree*) to 5 (*agree*). The scale consisted of the following subscales: ‘solidarity and proactiveness’ (three items), ‘self‐determination’ (three items), ‘sense of attachment’ (three items), and ‘reliance on others’ (three items). The internal consistency reliability of the scale was found to be acceptable (Cronbach's alpha coefficient = 0.837).^22^

### Analysis

After obtaining the descriptive statistics of the participants' characteristics, content analysis and multinomial logistic regression analysis were used to clarify their helping behaviours and examine the related factors, respectively.

#### Content analysis

We analysed the free‐answered data regarding experiences of helping or not being able to help older adults in the community using the content analysis method described by Funashima,^23^ based on Berelson's method.^24^ The questions for the analysis concerned (i) what difficulty the older adult faced (helping situations), (ii) how the participants helped older adults in situations in which they could help (helping behaviour), and (iii) why they could not help the older adult in the situations in which they could not help (reasons for not helping). We excluded cases of ‘informal planned helping behaviours’ by a family member or friend and ‘formal planned helping behaviours’ by a care professional or volunteer from the analysis with the intention of understanding spontaneous helping behaviours.

The following procedure was used to analyse the data for each question. First, the first author described the contents corresponding to the answers to the questions from each case (making a recording unit). Second, each recording unit was categorised based on its similarity in terms of content, and each category was named. Third, to examine the reliability of the categorisation, the second author categorised data on a sample of record units, which were randomly extracted from 20% of all record units, into the defined categories. The concordance rate between the two researchers was calculated using the method of Scott *et al.,* ^25^ and a criterion of 70% or above was set to ensure reliability.^23^

#### Logistic regression analysis

To understand the characteristics of community members who helped older adults, we performed multinominal logistic regression analysis to examine factors in the participants' characteristics related to helping behaviours. For this analysis, we classified each category of helping behaviour based on the content analysis into two types—a little helping and advanced helping—and used this as an outcome variable (i.e., no helping, a little helping, and advanced helping), and examined factors related to the implementation of a little helping and advanced helping, with reference to no helping.

The participants' ages were entered into the model using the forced entry method. We also entered sex, years of residence in the community, and whether they worked as health or social care professionals, had a family member with dementia, had experience with a PLWD other than a family member, knew the community general support centre, and had experience attending a dementia supporter program. Scores for dementia knowledge and the sense of community scale were entered using the forward stepwise selection method.

The significance level was set at <0.05 (two‐tailed). Statistical analysis was performed using SPSS Statistics version 22.0 for Windows.

### Ethical considerations

Survey responses were voluntary, and the respondents were given rewards by the survey company. Registrants under 18 years of age registered with their parents' consent. All data were anonymous, and informed consent was obtained from the online survey system. The study was conducted in accordance with the ethics guidelines of the Declaration of Helsinki. This study was approved by the Research Ethics Committee of the Graduate School of Medicine, University of Tokyo (#2019206NI).

## RESULTS

### Characteristics of participants

Table 1 shows the descriptive statistics of the participants' characteristics (*N* = 1000). The mean age (± standard deviation) was 44.6 (± 13.8) years, 50% were men, and approximately 10% were working as health/social care professionals. Only 6% of the participants had experience attending a dementia supporter program.

**Table 1:** Participant characteristics (*n* = 1000)

		All (*n* = 1000)		Experience of helping older adults					
				Yes (*n* = 441)		No (*n* = 559)			*P*‐value
		*n* (%)		*n* (%)		*n* (%)			
		Mean ± SD		Mean ± SD		Mean ± SD			
Age		44.6 ± 13.8		45.4 ± 13.8		44.0 ± 13.8			0.127
Sex, men		500 (50.0%)		193 (43.8%)		307 (54.9%)			0.001
Working as health/social care professionals		96 (9.6%)		63 (14.3%)		33 (5.9%)			<0.001
Education									
Junior high school		14 (1.4%)		3 (0.7%)		11 (2.0%)			0.178
High school		174 (17.4%)		74 (16.8%)		100 (17.9%)			
Junior or career college		222 (22.2%)		112 (25.4%)		110 (19.7%)			
University		514 (51.4%)		217 (49.2%)		297 (53.1%)			
Graduate school		70 (7.0%)		32 (7.3%)		38 (6.8%)			
Other		6 (0.6%)		3 (0.7%)		3 (0.5%)			
Living arrangement, living alone		239 (23.9%)		98 (22.2%)		141 (25.2%)			0.303
Years living in current community		16.8 ±15.5		16.8 ±15.4		16.8 ±15.6			0.950
Having family with dementia		279 (27.9%)		155 (35.1%)		124 (22.2%)			<0.001
Experience getting involved with person with dementia									
Involved continuously		86 (8.6%)		67 (15.2%)		19 (3.4%)			<0.001
Involved only once or for a short period of time		211 (21.1%)		126 (28.6%)		85 (15.2%)			
Never involved		703 (70.3%)		248 (56.2%)		455 (81.4%)			
Experience of attending dementia supporter program		60 (6.0%)		40 (9.1%)		20 (3.6%)			<0.001
Knowledge of dementia		8.4 ± 1.5		8.7 ± 1.3		8.1 ± 1.7			<0.001
Knowing community general support centre		353 (35.3%)		203 (46.0%)		150 (26.8%)			<0.001
Sense of community score		36.9 ± 5.3		37.7 ± 4.5		36.4 ± 5.7			<0.001

### Experience with helping or not helping older adults

Among the participants, 481 had had helping experiences while 241 had encountered an older adult in trouble but could not help. Among these 481 and 241 participants, 40 and 34 were excluded, respectively, because of a lack of details about the situations (eight and 12) or they described helping a family member/friend or as a care professional/volunteer (22 and 32). Therefore, the helping and not‐helping experiences of 441 (44.1%) and 207 (20.7%) participants, respectively, were analysed.

The concordance rates of the two researchers using Scott's method were 90.5% (helping situations), 90.0% (helping behaviour), 91.1% (not helping situations), and 84.9% (reasons not helping), indicating good overall concordance.

#### Situations in which participants encountered older adults in trouble

Table 2 shows the situations in which the participants encountered older adults in trouble in cases in which they helped (*n* = 441) and could not help (*n* = 207). The situations were classified into seven categories, comprising 18 subcategories. If multiple categories were applicable for each situation, each was counted.

**Table 2:** Situations in which the participants encountered older adults in trouble

		Able to support (*n* = 441)	Not able to support (*n* = 207)
		*n* (%)	*n* (%)
**1. Difficulty walking or moving**		**84 (19.1)**	**34 (16.4)**
Difficulty walking or moving		33 (7.5)	12 (5.8)
Difficulty carrying heavy baggage		51 (11.6)	22 (10.6)
**2. Difficulty standing in city or public transportation**		**32 (7.3)**	**8 (3.9)**
**3. Lost in a city or on public transportation (regardless of whether they had dementia)**		**153 (34.7)**	**64 (30.9)**
Being lost in the city		138 (31.3)	46 (22.2)
Being lost on public transportation		15 (3.4)	18 (8.7)
**4. Accidents and poor physical condition**		**80 (18.1)**	**47 (22.7)**
Falling		35 (7.9)	21 (10.1)
Having injuries/illness		32 (7.3)	19 (9.2)
Being in danger on roads or at railroad crossings		8 (1.8)	7 (3.4)
Going missing because of dementia		5 (1.1)	0 (0.0)
**5. Situations indicating possibility of dementia**		**24 (5.5)**	**28 (13.6)**
Unusual behaviour/appearance such as shouting or talking to oneself		17 (3.9)	26 (12.6)
Confused because of not understanding his/her own situation		7 (1.6)	2 (1.0)
**6. Difficulties in daily life**		**67 (15.3)**	**18 (8.6)**
Not being able to shop properly		14 (3.2)	3 (1.4)
Not being able to operate machines or follow procedures such as payment		22 (5.0)	9 (4.3)
Dropping or losing things or money		18 (4.1)	5 (2.4)
Not being able to use the toilet properly on the way out		3 (0.7)	1 (0.5)
Having trouble doing housework, eating, or going out		10 (2.3)	0 (0.0)
**7. Others**		**15 (3.4)**	**13 (6.3)**
Not understanding what kind of difficulty the older adult was having		0 (0.0)	5 (2.4)
Others		15 (3.4)	8 (3.9)

The boldface is the total in each category.

The most common situations in which older adults faced difficulties included being lost in the city or on public transportation, regardless of whether they had dementia (34.7% and 30.9% for cases in which participants helped and could not help, respectively). About 20% of participants encountered an older adult who had trouble walking or moving (19.1% and 16.4%, respectively) or had an accident or was in poor physical condition (18.1% and 22.7%, respectively).

‘Situations indicating the possibility of dementia’ frequently occurred in the same cases as older adults who were lost in the city/on public transportation or had had an accident, as mentioned above. More participants could not help (13.6%) than could help (5.5%) in situations with unusual behaviour or appearance, such as shouting or talking to themselves or being confused because they did not understand their own situations (Table 2).

#### Helping behaviours provided

Table 3 shows the helping behaviours provided by the participants in the cases where they helped older adults (*n* = 441). The most common helping behaviours were helping them walk or move, including carrying their baggage (20.0%), and taking care of them in accidents, such as checking their condition by talking to them or helping them get up (16.8%). More than 10% of the participants reported that they supported an older adult with difficulties in daily life (10.4%), gave directions to or accompanied an older adult to a destination (11.6% and 12.9%, respectively), and contacted police or an ambulance (11.8%). Some participants contacted store/station staff or family members (7.9%), while others offered their seats on transportation or their turn in line (7.3%; Table 3).

**Table 3:** Participants' helping behaviours (*n* = 441)

		*n* (%)
**1. Help walking/moving**		**88 (20.0)**
Assisting with walking/movement		32 (7.3)
Carrying their baggage		57 (12.9)
**2. Giving up a seat on public transit or a turn in line**		**32 (7.3)**
**3. Giving directions to a destination**		**51 (11.6)**
Showing the way to homes and other destinations		37 (8.4)
Providing information on train to destination		15 (3.4)
**4. Accompanying to destination**		**57 (12.9)**
**5. Taking care of older adult in an accident**		**74 (16.8)**
Checking older adult's conditions by talking to him/her		35 (7.9)
Helping older adult get up		26 (5.9)
Taking care of older adult		12 (2.7)
Searching for an older adult who has gone missing		5 (1.1)
**6. Contacting police or an ambulance**		**52 (11.8)**
Calling police		45 (10.2)
Calling an ambulance		7 (1.6)
**7. Contacting to staff or family member**		**35 (7.9)**
Contacting family members		18 (4.1)
Asking for help from store clerks		10 (2.3)
Asking for help from station staff		5 (1.1)
Calling a cab		2 (0.5)
Contacting municipality staff		1 (0.2)
**8. Support for difficulties in daily life**		**46 (10.4)**
Support for machine operation/procedures		19 (4.3)
Looking for or picking up things/money		15 (3.4)
Support for shopping		12 (2.7)
Assisting with housework, providing meals, going out		13 (2.9)
**9. Talking and watching over**		**23 (5.2)**
Talking to older adults		11 (2.5)
Accompanying older adults		8 (1.8)
Watching over them so that older adults can move without haste		4 (0.9)
**10. Others**		**10 (2.3)**

Since there are some cases involving multiple helping behaviours, the percentages do not sum to 100%.

The boldface is the total in each category.

#### Reasons for not being able to help

Table 4 shows the reasons why participants could not help older adults (*n* = 207). The most common reason was that they were in a situation that prevented them from helping (e.g., in a hurry to get somewhere, physically unable to help because they were driving or far away; 42.5%). About 20% did not feel a sense of responsibility to help the older adult (19.3%), either because someone else had already helped or they assumed someone else would help. Some said they did not know how to help older adults (17.4%) and hesitated to help (14.4%) because they did not want to get involved or were afraid of the older adult's unusual appearance (Table 4).

**Table 4:** Reasons for being unable to help (*N* = 207)

		*n* (%)
**1. Not able to determine the need for support**		**13 (6.3)**
**2. Not being responsible for providing help**		**40 (19.3)**
Another person already helping the older adult		23 (11.1)
Believing that another person will assist the older adult		11 (5.3)
No room in the participant's heart		4 (1.9)
Not knowing the older adult		2 (1.0)
**3. Hesitating to help**		**30 (14.4)**
Not wanting to get involved in any problems/difficulties		15 (7.2)
Being afraid because of unusual appearance		15 (7.2)
**4. Being in situations that result in not being able to help**		**88 (42.5)**
Being in a hurry		65 (31.4)
Physically unable to help because of driving a car or being far away		15 (7.2)
Not able to help by themselves in the situation		6 (2.9)
Not able to deal with it sufficiently despite trying to help		2 (1.0)
**5. Not knowing how to help**		**36 (17.4)**
Not able to talk to older adults well		25 (12.1)
Not knowing how to help		11 (5.3)
**6. Others**		**12 (5.8)**
Refused by the older adult		6 (2.9)
Difficulty resolved before helping		2 (1.0)
Others		4 (1.9)

### Factors related to helping behaviours

We classified the helping behaviours extracted from the content analysis into ‘a little help’ (e.g., giving up a seat on public transit, letting them move ahead in line, giving directions to a destination) and ‘advanced help’ (e.g., accompanying them to a destination, helping them walk/move, providing accident care, contacting police or an ambulance, contacting staff or a family member, providing support for daily living, talking with them or watching over them).

Table 5 shows the results of the multinomial logistic regression analysis, which was used to examine the associations between helping experience and participants' characteristics. The characteristics related to a little helping experience were fewer years of residence in the current community (adjusted odds ratio (AOR) = 0.97, *P* = 0.007) and higher scores for dementia knowledge (AOR = 1.28, *P* = 0.010). The characteristics related to advanced helping experience were older age (AOR = 1.01, *P* = 0.044), female (AOR = 1.52, *P* = 0.005), having a family member with dementia (AOR = 1.73, p = 0.001), having experience interacting with a PLWD other than a family member, continuous involvement/involvement for a short period of time (AOR = 4.99 and 2.52, respectively; *P* < 0.001), knowing about the community general support centre (AOR = 1.59, *P* = 0.004), higher scores for dementia knowledge (AOR = 1.17, *P* = 0.003), and higher scores for sense of community (AOR = 1.04, *P* = 0.001).

**Table 5:** Factors affecting helping behaviour (*n* = 1000)

	A little helping behaviour				Advanced helping behaviour			
	OR	95% CI		*P*‐value	OR	95% CI		*P*‐value
Age	0.98	0.96−1.00		0.054	1.01	1.00−1.03		0.044
Sex, female	1.12	0.67−1.89		0.661	1.52	1.13−2.04		0.005
Years of residence in the current community	0.97	0.94−0.99		0.007	1.00	0.99−1.01		0.726
Having a family member with dementia	0.57	0.28−1.16		0.120	1.73	1.26−2.38		0.001
Experience with person with dementia								
Involved continuously	2.20	0.66−7.27		0.198	4.99	2.80−8.90		<0.001
Involved only once or for a short period of time	1.65	0.88−3.10		0.119	2.52	1.76−3.61		<0.001
Knowing the community general support centre	0.71	0.36−1.39		0.318	1.59	1.16−2.19		0.004
Knowledge of dementia score	1.28	1.06−1.55		0.010	1.17	1.06−1.30		0.003
Sense of community score	1.01	0.98−1.05		0.556	1.04	1.02−1.06		0.001

Reference category: no experience in helping.

## DISCUSSION

This study investigated community members' experience of spontaneous helping behaviours by asking whether they had helped an older adult in trouble. The results showed that about 40% and 20% of participants had experience with providing help or not being able to do so. The multivariate analyses demonstrated that participants with helping experience had more experience involving PLWD, knowledge of dementia and community resources for supporting older adults, and a stronger sense of community. To our knowledge, this is the first study to clarify actual situations of community members across diverse age groups spontaneously helping older adults in the community. The results could provide insightful knowledge to promote community members' helping behaviours for older adults, contributing to the realisation of AFCCs.

The results of the content analysis showed that community members experienced a variety of spontaneous helping behaviours, from a little help on the spot (e.g., yielding seats or giving directions to a destination) to further in‐depth help (e.g., taking care of older adults in an accident, accompanying the older adult to their destination, contacting public and private agencies/family, support in daily life). Since little is known about community members' spontaneous helping behaviours towards older adults, the categories developed in our content analysis could be useful as a draft framework for future research on community members' helping behaviours.

The results of the multivariate analyses showed that there were differences in related factors among the types of helping behaviours. In the category of little help without specific knowledge or skills (e.g., yielding seats/turns, giving directions to a destination), fewer years of residence in the current community and higher dementia knowledge scores were the only associated factors. However, for in‐depth advanced helping behaviours, the associated factors were older age, being female, experience with PLWD, knowledge of dementia and general community support centres, and sense of community. There could be different strategies for promoting various helping behaviours. The various factors identified in this study could be useful for considering specific strategies, especially for in‐depth advanced helping behaviours.

The results showed that experience with interacting with PLWD or having a family member with dementia was associated with advanced helping behaviours. Meanwhile, more knowledge of dementia was associated with both a little helping behaviour and advanced helping behaviour. These results are consistent with previous studies that revealed the associations between knowledge/attitudes and the helping behaviours of community members towards PLWD.^7^, ^8^, ^10^ Our results also suggest that dementia knowledge and interactions with PLWD could promote in‐depth advanced helping behaviours in addition to attitude improvement by reducing dementia‐related stigma. While this study did not specifically investigate ageist attitudes or interactions with older adults without dementia, these could also influence helping behaviours and should therefore be investigated in future research.

In addition, a sense of community and knowledge of community resources (e.g., community general support centres) were also associated with advanced helping behaviours. This is consistent with previous studies that showed an association between people's sense of community and participation in volunteering,^17^, ^26^ a type of planned helping behaviour. Higher perceptions of community and knowledge of community resources might lead to a high awareness of support and willingness to provide advanced support for older adults.

The results for the associated factors could provide useful suggestions for promoting community helping behaviours for older adults. Providing educational programs that include elements of involvement with older adults (including PLWD) and approaches that foster perceptions of community could be effective. In a previous study, we developed and evaluated a dementia educational program for community members that included a short film illustrating the daily life of an older woman living with dementia and virtual reality movies from her perspective. The results showed an improvement in attitudes towards PLWD and the intention to help PLWD.^27^ Simulated experience involving PLWD could be effective for increasing community members' awareness of support. Another study indicated that small‐group workshops with discussions improved participants' psychological sense of community and their intention to engage in social actions such as volunteering.^28^ This suggests that including small‐group discussions in future educational programs could help foster people's awareness of community and its resources, thus leading to advanced helping behaviours.

Our content analyses of reasons for not being able to help an older adult could also provide insightful suggestions. Aside from being physically unable to help, common reasons for not being able to help were that they could not determine the need for support, did not feel responsible for helping, felt hesitant to help, or did not know how to help (6%–19%). These elements are partially consistent with Latane and Darley's^29^ decision model of helping. Overcoming such barriers requires training on responsibility for helping and how to provide help. Gaesser *et al*. revealed that when presented with a situation depicting another person's plight, participants who imagined an event in which they helped the person (episodic simulation^30^) showed increased prosocial intentions.^31^ In our previous study, a dementia educational program using a simulation card game was effective for improving attitudes towards PLWD.^32^ Such simulation training designed to evoke an image of helping could be effective for motivating and teaching helping behaviours.

This study has several limitations. First, since the web survey was conducted for community members registered with the survey company and living in certain municipalities of the Tokyo Metropolis, the generalisability of the results is unknown. For example, it is possible that the subjects who voluntarily responded to the web survey performed advanced helping behaviours more frequently than community members as a whole. Also, community members in rural areas might provide advanced helping that is unique, but it might not be clarified in this study. Second, this study might not have identified all cases in which the participants could or could not help. For the cases in which they could help, they were asked to select the most memorable cases. However, there might have been other cases in which they provided help. For those not able to help, there might be difficult situations for older adults that the participants were unaware of. Third, since this was a cross‐sectional study, the causal relationships were unclear. In the future, longitudinal studies should be conducted to examine whether specific training for community members can promote helping behaviours towards older adults.

## CONCLUSION

Community members provided older adults with various types of spontaneous help, including help with walking, accident care, giving directions to or accompanying them to a destination, and support in daily life. Having a family member with dementia, experience interacting with a PLWD, knowledge of dementia and community general support centres, and a stronger sense of community were associated with advanced helping behaviours. Educational interventions for community members, including interactions with older adults, including PLWD, a focus on community perceptions, and training in actual helping actions could effectively promote helping behaviours in the community, leading to the construction of effective AFCCs.

## FUNDING INOFRMATION

The authors disclosed receipt of the following financial support for the research, authorship, and/or publication of this article: This work was supported by JSPS KAKENHI (Grant Number JP19H03956). This study was approved by the ethics committee of the University of Tokyo (No. 2019206NI).

## Data Availability

Research data are not shared.
